# Diagnostic accuracy of the iNPH Radscale in idiopathic normal pressure hydrocephalus

**DOI:** 10.1371/journal.pone.0232275

**Published:** 2020-04-24

**Authors:** Karin Kockum, Johan Virhammar, Katrine Riklund, Lars Söderström, Elna-Marie Larsson, Katarina Laurell

**Affiliations:** 1 Department of Clinical Science, Neurosciences, Umeå university, Östersund, Sweden; 2 Department of Neuroscience, Neurology, Uppsala University Hospital, Uppsala, Sweden; 3 Department of Radiation Sciences, Diagnostic Radiology, Umeå University, Umeå, Sweden; 4 Unit of Research, Education and Development, Östersund Hospital, Östersund, Sweden; 5 Department of Surgical Sciences, Radiology, Uppsala University, Uppsala, Sweden; Goethe University Hospital Frankfurt, GERMANY

## Abstract

**Background and purpose:**

The idiopathic normal pressure hydrocephalus (iNPH) Radscale was developed to standardize the evaluation of radiological signs in iNPH. The purpose of this study was to estimate the diagnostic accuracy of the iNPH Radscale in a sample of “true positive” and “true negative” cases.

**Methods:**

Seventy-five patients with definite iNPH, i.e. who had improved at clinical follow-up one year after ventriculoperitoneal shunt surgery, were compared with 55 asymptomatic individuals from the general population. A radiologist assessed the seven radiological features of the iNPH Radscale in computed tomography of the brain in the patients (preoperatively) and controls.

**Results:**

The iNPH Radscale score was significantly higher in the iNPH group (Median = 10, interquartile range 9–11) than in the control group (Median = 1, interquartile range 1–2) (p <0.001). Receiver operated characteristics analysis yielded an area under the curve of 99.7%, and an iNPH Radscale score ≤ 4 identified those without iNPH, with a sensitivity of 100%, specificity of 96% and overall accuracy of 98.5%.

**Conclusions:**

In this study, iNPH Radscale could accurately discriminate between patients with definite iNPH and asymptomatic individuals over 65 years old. According to the results, a diagnosis of iNPH is very likely in patients with an iNPH Radscale score above 8 and corresponding clinical symptoms. On the other hand, the diagnosis should be questioned when the iNPH Radscale score is below the cut-off level of 4. We conclude that the iNPH Radscale could work as a diagnostic screening tool to detect iNPH. Whether the scale also can be used to predict shunt outcome needs further studies.

## Background

Idiopathic normal pressure hydrocephalus (iNPH), is a neurological condition affecting gait, balance, cognition and continence [1]. The disease is more common than previously expected, with a prevalence of 3.7% for those over 65 years old, and is increasing with age [2]. The clinical symptoms of iNPH may overlap with those of other neurological conditions such Alzheimer’s disease and Parkinson disease. The diagnostic guidelines for iNPH are founded on typical symptoms combined with radiological findings [3, 4]. To select candidates for shunt surgery, supplemental tests, such as the tap-test, are often used [5].

The importance of identifying and correctly diagnose iNPH patients is underlined by the fact that up to 80% improve after treatment with a ventriculoperitoneal shunt [6].

Symptom scales have been launched in order to structure the clinical assessment, and to allow comparison of symptoms over time and between sites [7, 8]. The iNPH Radscale [9] was introduced as a screening tool to structure the assessment of radiological features associated with iNPH and includes mandatory and supportive radiological parameters listed in both diagnostic guidelines [3, 4]. The seven parameters of the scale derive from previous research [10–14]. The classical radiological findings of iNPH include ventriculomegaly with enlarged lateral ventricles and temporal horns, displacing the corpus callosum upwards towards the falx with a sharp callosal angle and narrowed parietal sulci at the vertex while the Sylvian fissures are enlarged. The combination of ventriculomegaly, narrowing of high parietal sulci and widened Sylvian fissures is called DESH (disproportional enlarged sulci hydrocephalus), and is a key feature in the Japanese guidelines [3, 15]. When possible, the iNPH Radscale uses quantitative variables and cut-off levels, in order to objectify and facilitate the interpretation of the radiological findings, see online supplement for image atlas with corresponding cut-off levels. Thus, the iNPH Radscale could serve as a checklist for the radiologist for evaluation and grading of the images.

In previous studies, the iNPH Radscale score was associated with symptom severity [9] and could be used in both computed tomography (CT) and magnetic resonance imaging (MRI), with substantial to almost perfect concordance between investigators and modalities [16].

As a next step, this study aims to evaluate the diagnostic accuracy of the iNPH Radscale in patients with definite iNPH and asymptomatic individuals over 65 years old and to find suitable cut-offs, determined by the sensitivity and specificity of the scale.

## Methods

Regional Ethical boards in Uppsala and Umeå approved of the study. Approval number: Uppsala 2015/174. Umeå 2014/180-31. The iNPH patients did not sign individual informed consent since this was a retrospective study and all data analyzed were collected as part of routine care. The asymptomatic participants gave written informed consent to have data from their medical records used in research.

The material consisted of a clinical sample of 75 definite iNPH patients (48 men and 27 women, median age 74 years (interquartile range (IQR) 70–77) and 55 asymptomatic individuals (24 men and 31 women, median age 70 years (IQR 69–73) from the general population.

The iNPH patients derived from Uppsala University hospital, where 332 patients with suspected iNPH received shunts during 2011–2015, previously described [17].

To be included in the study as a definite iNPH, the following criteria needed to be fulfilled:

1. Preoperative brain CT within eight days prior to surgery, with maximum 1 mm slice thickness stored in the picture archiving and communication system (PACS)
2. Clinical data from one-year follow up
3. Improvement in symptoms of the treatment

A positive shunt response as defined below (a—c):

- A 20% reduction in the time or the number of steps in at least one of the two motor function tests (Timed Up and Go, and 10 m walk)
- Four levels increase in the Mini-Mental State Examination
- One level increase in the continence scale and two levels increase in the Mini-Mental State Examination

This definition of a positive shunt response has been used previously by our group (8).

**Fig 1:**
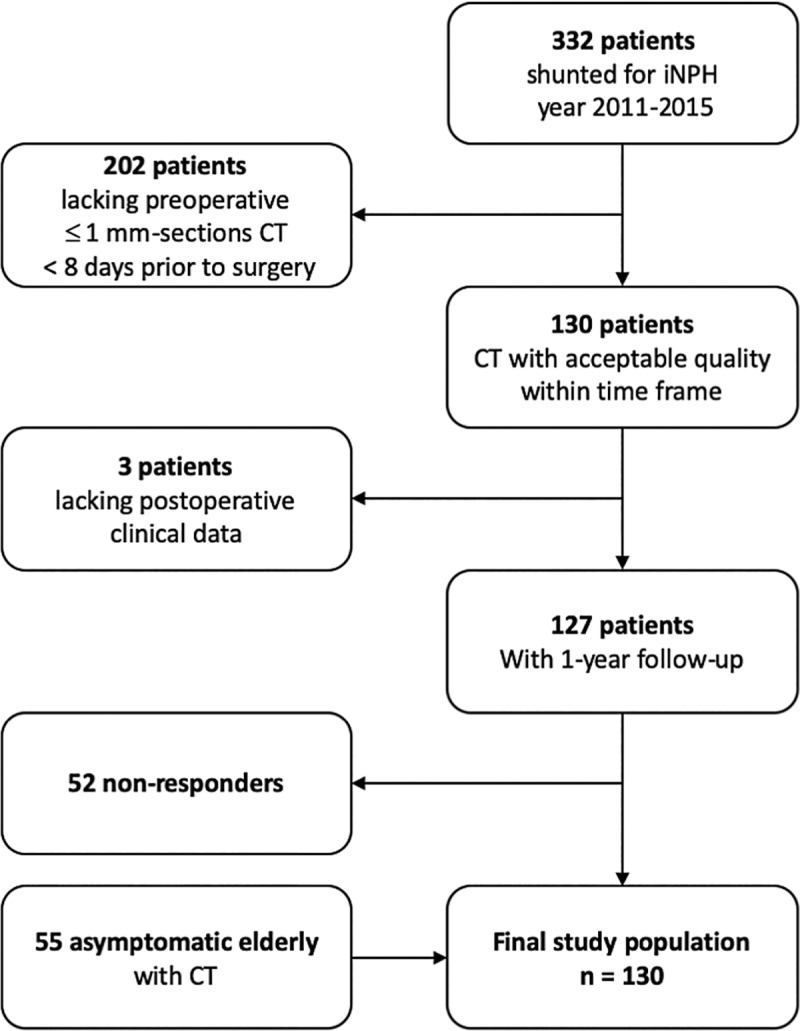
Flow chart of patient selection.

See Fig 1 for flow chart of patient selection.

The final sample of 75 patients with definite iNPH all had ventriculomegaly and clinical signs of iNPH, i.e. impaired gait in combination with cognitive deficiency and/or incontinence symptoms. The median symptom duration was 26 months (IQR 17–48). The diagnostic evaluation included multidisciplinary clinical assessment, infusion test and CSF tap-test. Biomarkers of CSF were analyzed for differential diagnoses such as Alzheimer’s disease.

The control group consisted of 55 individuals, 65 years or older from the general population, who participated in a previous study on the prevalence of iNPH [2]. Those without symptoms of iNPH, defined as at least 90 points on the iNPH symptom scale [7] were included in the present study, regardless of findings on a non-contrast enhanced brain CT. No lumbar puncture was performed as it was considered too invasive for volunteers from the general population.

The total sample thus consisted of 130 individuals, with significantly more men in the definite iNPH group (n = 75) compared to the control group (n = 55) (p = 0.005) while the number of women had similar distribution in both groups (p = 0.60). The patients with definite iNPH were significantly older than the controls (p = 0.02).

A specialist in diagnostic radiology (first author), blinded to clinical data, assessed all images according to the iNPH Radscale. Seven radiological parameters were included; Evans’ index [10] and the width of the temporal horns were measured to assess ventriculomegaly. Morphology of the sulci were assessed by narrowing of sulci, focal dilation of sulci and width of the Sylvian fissures. Further, the callosal angle was measured as described by Ishii [18] and periventricular white matter changes assessed. See the online supplement for atlas of included parameters with cut-off values for each scoring level.

Ethical approval was obtained from the Regional Ethical boards in Uppsala and Umeå (Dnr 2015/174 and 2014/180-31).

## Statistics

Sample characteristics were described using median and interquartile range (IQR). Differences between groups were calculated using Mann-Whitney U-test or Chi^2^-test when applicable. The index test”iNPH Radscale” score was compared to the reference standard”shunt response” using receiver operating characteristics curve, to yield a suitable cut-off between the groups and to calculate the area under the curve. The diagnostic validity of the iNPH Radscale was assessed by calculating sensitivity and specificity, with 95% confidence intervals (95% CI) to estimate the precision. Overall accuracy and likelihood ratio were calculated from the sensitivity and specificity, at the chosen cut-off of ≤ 4. Likelihood ratios where used instead of predictive values, as a prevalence independent equivalent. Simple logistic regression was used to assess the probability of having a definite iNPH diagnosis at different iNPH Radscale scores. Goodness of fit was assessed by Hosmer and Lemeshow test. Significance level was set to 0.05. All calculations were performed using SPSS version 25 (IBM Corp, Armonk, NY, USA).

## Results

**Fig 2:**
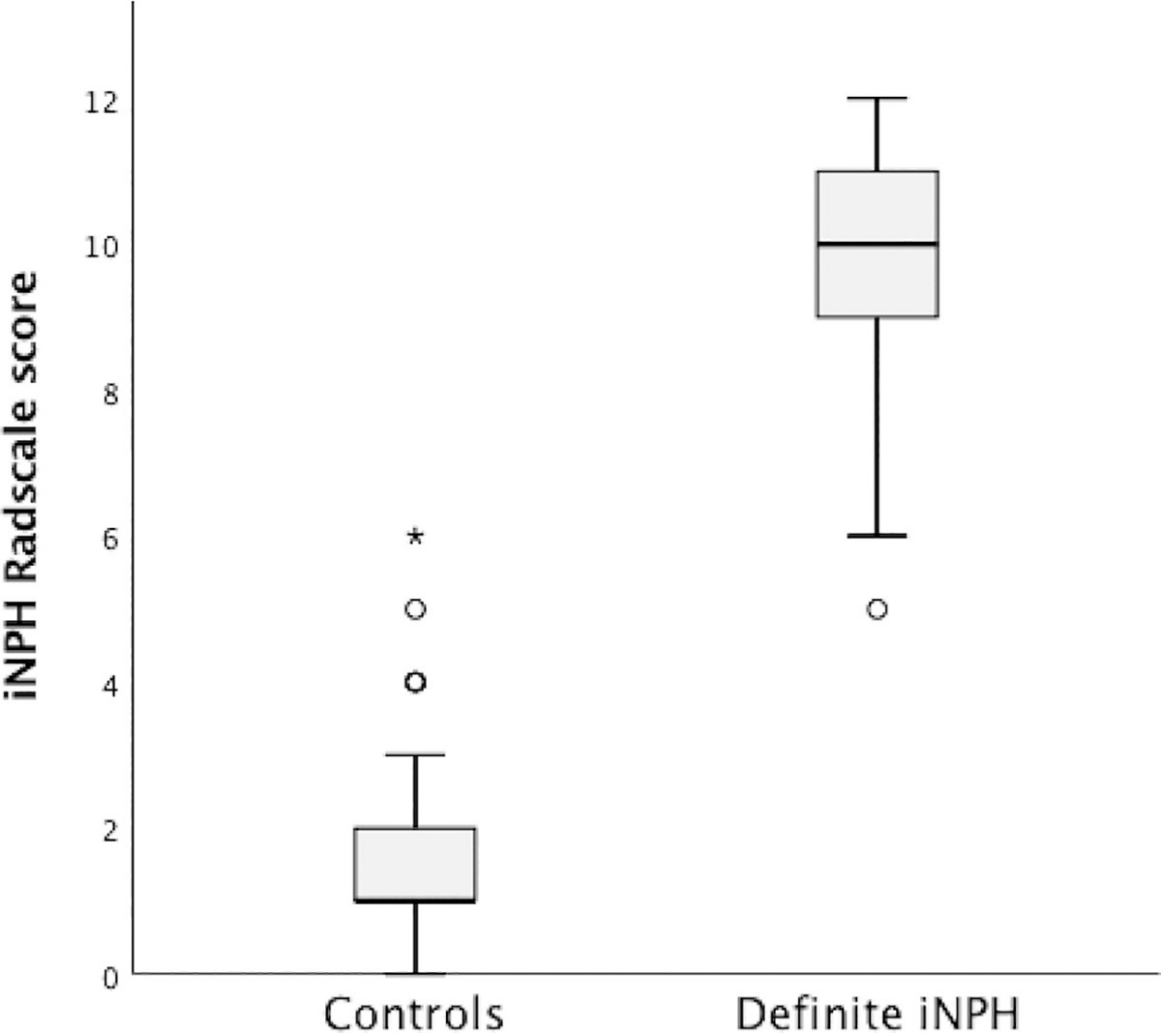
Box plot of iNPH Radscale score. The distribution of scores for the definite iNPH and control group, respectively.

The median iNPH Radscale score was significantly higher (Md = 10, IQR 9–11) in the definite iNPH group than in the control group (Md = 1, IQR 1–2) (p <0.001), see Fig 2.

**Fig 3:**
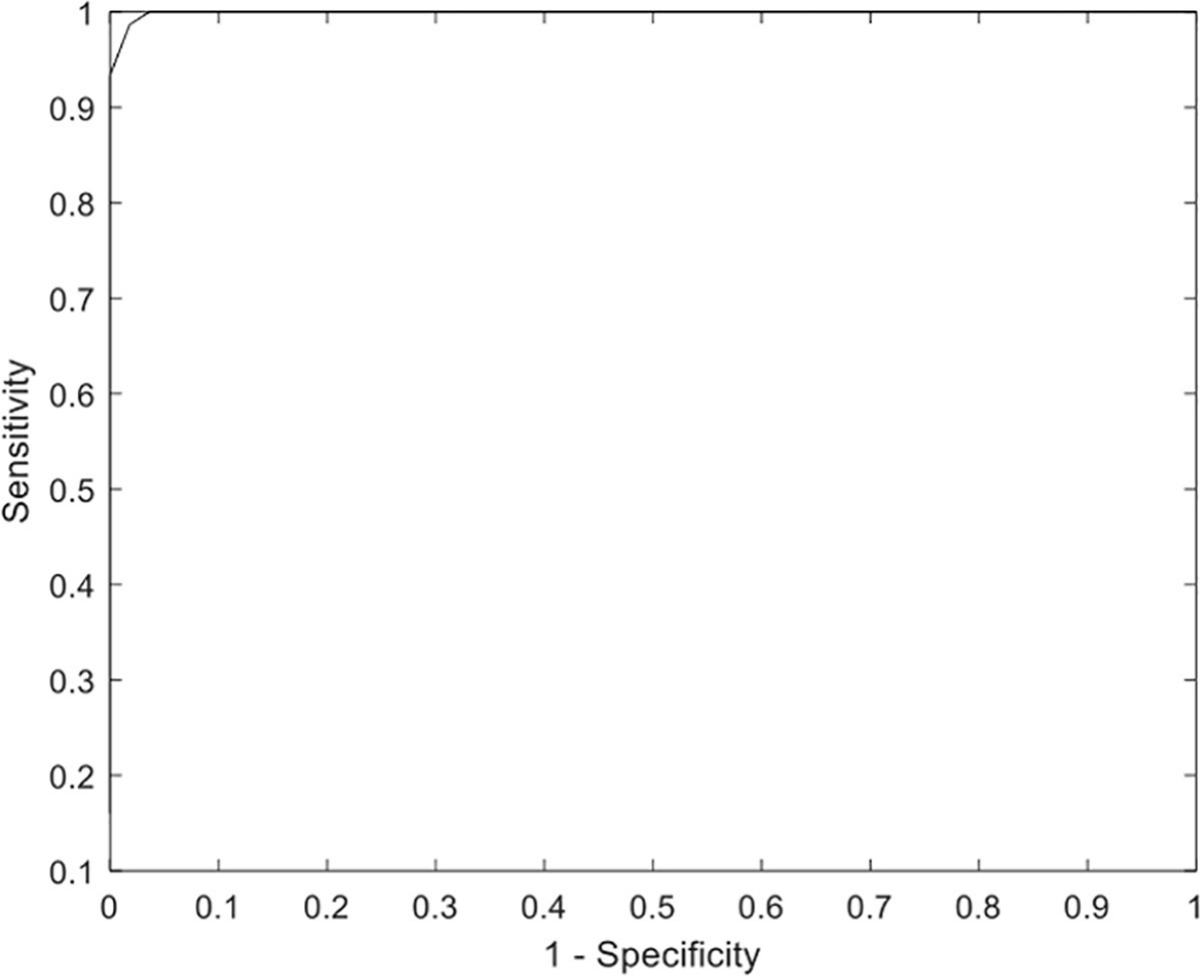
Receiver operating characteristic analysis. The sensitivity and 1 – specificity of iNPH Radscale for the definite iNPH group and the control group.

Receiver operating characteristic analysis yielded an area under the curve of 99%, when plotting the iNPH Radscale score against definite iNPH and controls, see Fig 3.

The sensitivity and specificity for the different iNPH Radscale scoring levels are summarized in Table 1.The optimal cut-off level for separating those with definite iNPH from asymptomatic controls was 4, with a sensitivity of 100% (95% CI 95.2–100) and specificity of 96.4% (95% CI 87.5–99.6). Cross tabulation of the iNPH Radscale score of ≤4 and > 4, for those with definite iNPH and for those not having the disease (controls), is illustrated in Table 2. The overall accuracy was calculated to 98.5% (95% CI 94.6–99.8), negative likelihood ratio to 0 and positive likelihood ratio to 11.

**Table 1:** Sensitivity and 1 – specificity for each scoring level of the iNPH Radscale.

iNPH Radscale	Sensitivity	1—Specificity
0	1.000	1.000
1	1.000	0.891
2	1.000	0.491
3	1.000	0.200
4	1.000	0.091
5	1.000	0.036
6	0.987	0.018
7	0.933	0.000
8	0.893	0.000
9	0.773	0.000
10	0.600	0.000
11	0.307	0.000
12	0.160	0.000

**Table 2:** Crosstabulation of definite iNPH by Radscale score.

		Definite iNPH	
		No (n)	Yes (n)
**iNPH Radscale**	≤ 4	53	0
	> 4	2	75

The two groups are separated by the cut-off of 4 points

**Fig 4:**
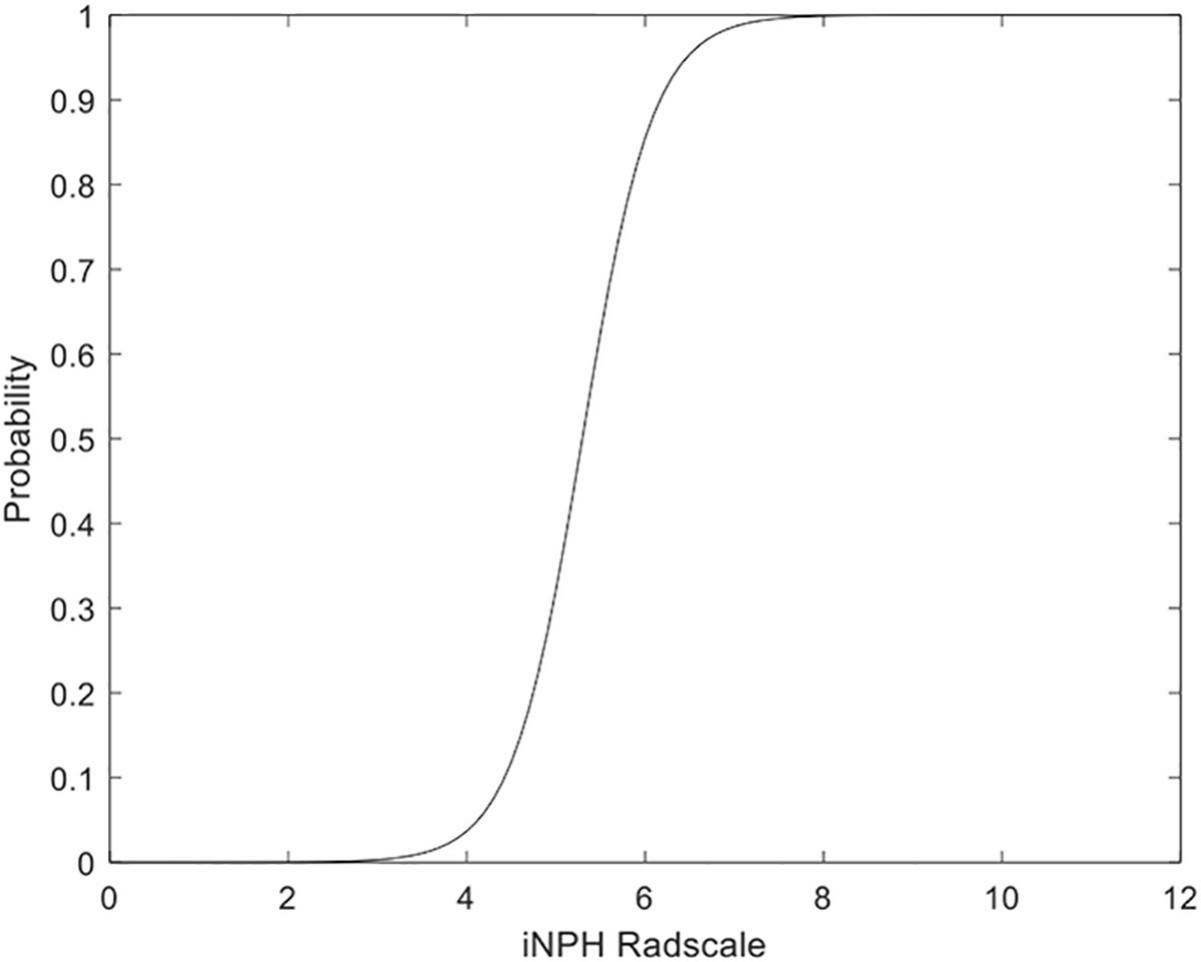
Fitted predicted probability plot. Each iNPH Radscale score is plotted against the fitted predicted probability from the logistic regression analysis.

In logistic regression analysis, there was a significant relationship between the iNPH Radscale and definite iNPH (Odds ratio 12.5, CI 95% 1.5–105.6, p = 0.02). The model shows goodness of fit (Hosmer and Lemeshow Chi^2^ 0.147, 7 degrees of freedom, p = 1). Fig 4 illustrates that low iNPH Radscale scores indicate low probability of iNPH and vice versa, i.e. that an iNPH Radscale score ≤ 4corresponds to a risk for iNPH of less than 4% and scores ≥ 8 suggest high probability of the disease.

## Discussion

In this study, the iNPH Radscale discriminated definite iNPH patients from asymptomatic individuals over 65 years with a high diagnostic accuracy. A cut-off level of 4 corresponds to 100% sensitivity and 96% specificity, under this score the negative likelihood ratio was 0, suggesting that iNPH could be ruled out. A Radscale score ≥ 8 suggests high probability of iNPH, if typical symptoms are present, with a positive likelihood ratio of 11. In the intermediate group with 5–7 scores on the iNPH Radscale, the patients should be further evaluated for iNPH depending on symptoms, comorbidity and results of additional investigations.

Several previous studies have described the prognostic value of imaging features in predicting shunt response in iNPH patients [14, 19–22], but only a few studies have focused on the diagnostic value [18, 23, 24]. One such example is the study by Ishii et al [18], who showed that the combination of a callosal angle of less than 90° and an Evans’ index over 0.30 could separate probable iNPH from Alzheimer disease. Likewise, Miskin et al. [24] demonstrated that Evans’ index combined with callosal angle could separate definite iNPH from healthy controls as well as Alzheimer’s disease. Our results are in line with these publications and support that imaging is crucial in the diagnosis of iNPH. However, the observation in the present study that the iNPH Radscale discriminates between definite iNPH and asymptomatic individuals does not tell whether the scale can be used to predict the outcome of surgery as well. The indication for surgery must be evaluated separately, taking age, comorbidity and other prognostic factors into consideration [17, 25].

The iNPH Radscale can be used on CT or MR scans when iNPH is suspected by the clinicians, or in *en passant* finding of ventriculomegaly for example in CT scans after fall accidents. In the latter case, it is suitable to add the suspicion of iNPH in the radiological report, to be further correlated to the patient’s symptoms. The combination of a high iNPH Radscale score and prominent symptoms is highly suggestive of iNPH. Detecting iNPH at an early stage is important as treatment is less beneficial in later stages of the disease [26].

There are some weaknesses as well as strengths that need to be highlighted:

First, the shunt candidates were selected in clinical practice, in which assessment of imaging of the brain was included which could have led to incorporation bias. However, the iNPH Radscale was neither used in the surgical selection process nor in the definition of definite iNPH. Further, the controls were selected merely from the lack of symptoms, before imaging was evaluated, which is a strength of the study.

Second, the radiologist was not blinded to whom had received a shunt. However, since several of the measurements were quantitative and unlikely to be influenced by knowledge of clinical presentation of the patient this weakness should be of less importance. In the control group, on the other hand, the radiologist was blinded to all clinical data.

Third, the responders were slightly older, and men were overrepresented. Since the prevalence of iNPH increase with age, this is not surprising [27]. However, it is interesting to speculate why men are overrepresented in the shunted group. There are indications that iNPH is more common in men [2], but it could also be influenced by gender differences in patient selection for surgery as well as in the patients’ health care seeking behavior.

In a clinical setting, it is of great value to be able to rule out iNPH and define who is highly likely to suffer from iNPH. The result of this study supports that the iNPH Radscale is a valid test with no overlap between true positive and true negative cases. The iNPH Radscale is rather simple to use, where the scoring usually takes under five minutes including reangluation of the coronal plane for callosal angle. The test is non-invasive and demonstrates in this study high sensitivity, and thus could be useful in the diagnostic toolbox for iNPH.

Previous studies have shown different results whether radiological features have a prognostic value or not in identifying shunt responders, and such studies are warranted for the total iNPH Radscale as well. Further, future research should evaluate whether the iNPH Radscale also can be used to differentiate between iNPH and other conditions of impaired gait and cognition such as Alzheimer’s and Parkinson’s disease.

## Conclusion

In this study, the iNPH Radscale could accurately discriminate between patients with definite iNPH and asymptomatic individuals over 65 years of age. According to the results, a diagnosis of iNPH is very likely in patients with an iNPH Radscale score above 8 and corresponding clinical symptoms. On the other hand, the diagnosis should be questioned when the iNPH Radscale score is below the cut-off level of 4. We conclude that the iNPH Radscale seem to work as a diagnostic screening tool to detect iNPH. Whether the scale also can be used to predict shunt outcome needs further studies.

## Supporting information

S1 Fig iNPH Radscale atlas. Image examples and cut-off levels for each scoring level for the seven features included in the scale.
(PDF)
